# Transcriptome Analysis in Vulvar Squamous Cell Cancer

**DOI:** 10.3390/cancers13246372

**Published:** 2021-12-19

**Authors:** Katharina Prieske, Malik Alawi, Anna Jaeger, Maximilian Christian Wankner, Kathrin Eylmann, Susanne Reuter, Patrick Lebok, Eike Burandt, Niclas C. Blessin, Barbara Schmalfeldt, Leticia Oliveira-Ferrer, Simon A. Joosse, Linn Woelber

**Affiliations:** 1 Department of Gynecology and Gynecologic Oncology, University Medical Center Hamburg-Eppendorf, 20246 Hamburg, Germany; a.jaeger@uke.de (A.J.); k.eylmann@uke.de (K.E.); s.reuter@uke.de (S.R.); b.schmalfeldt@uke.de (B.S.); ferrer@uke.de (L.O.-F.); lwoelber@uke.de (L.W.); 2 Mildred Scheel Cancer Career Center HaTriCS4, University Medical Center Hamburg-Eppendorf, 20246 Hamburg, Germany; s.joosse@uke.de; 3 Colposcopy Clinic at the Jerusalem Hospital Hamburg, 20357 Hamburg, Germany; 4 Bioinformatics Core, University Medical Center Hamburg-Eppendorf, 20246 Hamburg, Germany; m.alawi@uke.de; 5 Department of Tumor Biology, University Medical Center Hamburg-Eppendorf, 20246 Hamburg, Germany; maximilian.wankner@semmelweis-hamburg.de; 6 Institute of Pathology, University Medical Center Hamburg-Eppendorf, 20246 Hamburg, Germany; p.lebok@uke.de (P.L.); e.burandt@uke.de (E.B.); n.blessin@uke.de (N.C.B.)

**Keywords:** vulvar cancer 1, sequencing 2, transcriptome 3, TP53 4, HPV 5, RNA 6

## Abstract

**Simple Summary:**

The number of women, especially younger women, diagnosed with vulvar cancer, has been rising mainly due to the infection with human papilloma virus (HPV) over the last years. In contrast to other tumor entities, limited information on the underlying genetic changes is available, and thus treatment advances, especially the development of personalized treatments, are hampered. We aimed to explore the RNA expression profiles in a group of 24 vulvar cancer samples in order to detect potential prognostic markers and therapeutic targets in order to establish to a more profound understanding of vulvar cancer carcinogenesis.

**Abstract:**

To date, therapeutic strategies in vulvar squamous cell carcinoma (VSCC) are lacking molecular pathological information and targeted therapy hasn’t been approved in the treatment of VSCC, yet. Two etiological pathways are widely accepted: HPV induced vs. HPV independent, associated with chronic skin disease, often harboring *TP53* mutations (mut). The aim of this analysis was to analyze the RNA expression patterns for subtype stratification on VSCC samples that can be integrated into the previously performed whole exome sequencing data for the detection of prognostic markers and potential therapeutic targets. We performed multiplex gene expression analysis (NanoString) with 770 genes in 24 prior next generation sequenced samples. An integrative data analysis was performed. Here, 98 genes were differentially expressed in TP53mut vs. HPV+ VSCC, in the TP53mut cohort, where 56 genes were upregulated and 42 were downregulated in comparison to the HPV+ tumors. Aberrant expression was primarily observed in cell cycle regulation, especially in HPV+ disease. Within the TP53mut group, a distinct cluster was identified that was correlated to a significantly worse overall survival (*p* = 0.017). The RNA expression profiles showed distinct patterns with regard to the known VSCC subtypes and could potentially enable further subclassification in the TP53mut groups

## 1. Introduction

Over the last decades, the incidence of vulvar squamous cell carcinoma (VSCC) has continuously been rising [1,2,3]. Two etiological pathways are widely accepted [4,5]. Approximately 40% of VSCCs are attributable to high-risk human papillomavirus (HPV), occurring more frequently in younger women [6]. The majority of VSCCs develop in postmenopausal women, are HPV independent, and are initiated by *TP53* mutations. These tumors develop from chronic dystrophic skin disease, mostly lichen sclerosus as a facultative precancerous condition. A third pathway causing VSCC, which arises independently of HPV infection or *TP53* mutations, has been proposed but is currently poorly understood [7,8]. While primary treatment comprises surgery with radical local excision of the primary tumor and surgical staging of the groins with or without adjuvant chemoradiation in case of advanced nodal involvement [9], therapeutic options are extremely limited in the case of surgically non-resectable recurrent VSCC or occurrence of distant metastases [10]. Treatment strategies, such as antiangiogenetic treatment or immunecheckpoint inhibition, are then generally experimental and adapted from cervical, anal, or head and neck cancer (HNSCC) [11,12,13]. Thorough molecular characterization, as performed by The Cancer Genome Atlas (TCGA) research network, with large scale genomic, epigenomic, transcriptomic, and proteomic analyses has deepened our understanding of various types of human cancers and has led to more accurate subtype stratification and prognoses. In many cases, potential new targets have led to investigation in clinical trials and changed the way patients are treated in the clinic. Unfortunately, vulvar cancer has not been considered by the TCGA and comparable molecular analyses are still lacking. It is intuitive that deep depth molecular characterization is a prerequisite for the characterization of potential targets in clinical trials and for improved treatment strategies in the long term.

A couple of groups have investigated the underlying genetic alterations in VSCC, mostly via next generation sequencing (NGS) panel analysis [14,15,16,17], two groups including our own, applied whole exome sequencing (WES) [5,18]. Due to huge differences in methodology, HPV detection, and sample size, the comparison was hampered. However, beyond *TP53* as a key oncogenic driver, alterations of the prooncogenic *PI3K/AKT/mTOR* pathway, including *HRAS, KRAS, PIK3CA, KMT2D,* and *PTEN* mutations, have consistently been reported across different studies. In addition, mutations in potential regulators of the *PI3K/Akt/mTOR* pathway like *FBXW7*, *NBPF1*, and *TSC2* were found, as well as in WES and hotspot analyses [5,8,18]. Han et al. reported *PI3KCA* mutations, but also high numbers of copy number gains, leading to a *PIK3CA* alteration rate of 60% in their HPV positive cohort. Recently, methylation profiling was performed in a set of 18 primary VSCC, including 3 HPV+ samples and compared to normal tissue [19]. Most of the genes that were found to be hypermethylated in VSCCs (e.g., ZSCAN1, ZNF135, ZNF471, or TBX3) are involved in transcription regulator activity.

While DNA sequencing can identify important drivers for malignant transformation of the cell, it insufficiently describes its actual phenotype. Transcriptome profiling has led to a more profound molecular understanding of carcinogenesis, expanding on the knowledge of distinct prognostic relevant subtypes, biomarker development, and the impact of the tumor microenvironment [20,21].

So far, very few groups have performed RNA expression analysis in VSCC. In a small set of five VSCC samples with matched normal controls, aberrant regulation of cell cycle, growth, and proliferation; cell death; and cellular development were detected. Recently, another transcriptome analysis of 23 VSCC and one control vulvar tissue was performed. Two distinct groups, defined by HPV status, with several differentially expressed genes (e.g., STMN1, FOXO6, FCGBP DMBX1, and GBX1) were detected. In the pathway analysis, enrichment for cytokine receptor and inflammatory signaling was revealed in the HPV+ tumors [22,23].

The aim of this analysis is to combine genomic and transcriptomic information in order to identify prognostic markers, deregulated pathways, and potential targets for new treatment strategies in VSCC. Therefore, 24 of the previously sequenced samples were subjected to multiplex gene expression analysis, including 770 genes.

## 2. Materials and Methods

### 2.1. Patients and Tumor Tissue

The tissue of 24 fresh frozen VSCC samples that had previously been subjected to WES were available [5]. Somatic variants from our WES analysis in vulvar cancer are provided in Table S1. Tissue samples were obtained at the University Medical Center Hamburg-Eppendorf during surgeries performed between 1998 and 2011. All samples were immediately snap frozen and stored in liquid nitrogen at −196 °C. Every sample was assessed on cryo-cut sections stained with hematoxylin and eosin. If necessary, the stromal parts were removed by scraping them using a scalpel to obtain at least 60% tumor cells in the sample used for RNA extraction. Pathological studies were carried out by Dr. Lebok, who is a specialized gynecopathologist. All of the patients enrolled gave written informed consent to access their tissue and review their clinical records, according to our investigational review board and ethics committee guidelines. (Ethics Committee of the Medical Board Hamburg reference number 190504). Data were retrieved from patient records and the institutional database providing information on clinicopathologic factors, and histology and therapeutic approaches. For tumor staging, the International Federation of Gynecology and Obstetrics (FIGO) stage groupings and the International Union against Cancer (UICC) tumor-node-metastasis (TNM) classification sixth edition were used for homogeneity [24,25].

### 2.2. Subgroup Definition

In our previous WES analysis of the present VSCC samples, *TP53* mutation status and HPV E7 integration into the human genome were analyzed. The tumors that showed neither *TP53* mutations nor HPV E7 integration were categorized as “double negatives” (OTHER).

### 2.3. RNA Extraction

Here, 20–30 cryosections (approximately 16 µm) were disintegrated using Precellys homogeniser (WVR International GmbH, Darmstadt, Germany). Subsequently, RNA was extracted using the RNAeasy Kit (Qiagen GmbH, Hilden, Germany), according to the manufacturer’s instructions. RNA quantity and integrity were assessed using a Bioanalyzer device (Agilent, Santa Clara, CA, USA). Then, 200–400 ng RNA was requested for nanostring analysis, depending on the RNA quality. The range of RIN values was >2.3–9.8 and the bionalyzer concentration ranged from 156–1436 ng/mL.

### 2.4. Sequencing and Bioinformatic Analysis

Multiplex gene expression analysis (NanoString CancerPath C2535) with 770 genes was performed in 24 of the 34 formerly sequenced VSCC samples (Table S2: Results of the differential expression analysis (TP53mut vs. HPV+). Integrative data analysis was carried out by the Bioinformatics Core of the University Medical Center Hamburg-Eppendorf.

Normalization was performed using RUVseq [26], as described previously by Bhattacharya and colleagues [27], where 38 of 40 housekeeping genes (*NUBP1* and *PRPF38A* were excluded) and of 4 of 6 positive controls (*ERCC_00035.1* and *ERCC_00034.1* were excluded) were selected as a reference group for the normalization process. One dimension of unwanted variation was removed (k = 1). Differential expression analysis was carried out with DESeq2 [27]. A gene was considered differentially expressed if the corresponding absolute log2-transformed foldchange (log2FC) was not less than 1 and, in addition, the false discovery rate (FDR) did not exceed the value of 0.1 The detection of the pathways over-represented in the set of differentially expressed genes was performed using ClusterProfiler [28], in combination with the Reactome Pathway Database [29].

### 2.5. Statistical Analysis

Statistical computations were executed using the R programming language (Foundation for Statistical Computing) and the free, online statistical tools from In-Silico Online, version 2.3.1 [30]. To test for associations between clinic-pathological risk factors and patient groups, cross tables were constructed and analyzed using the G-test with Williams’ correction. Median differences between patient groups were tested using the Wilcoxon rank test. Survival analyses were performed using Kaplan−Meier estimates accompanied with the log rank test, as well as the cox proportional hazard function for multivariable testing. Normalized gene expression counts were analyzed by unsupervised hierarchical clustering using Ward D2 linkage and Euclidian distance. For all of the statistical tests, significance was considered when *p* < 0.05.

## 3. Results

### 3.1. Subgroup Definition

**Figure 1:**
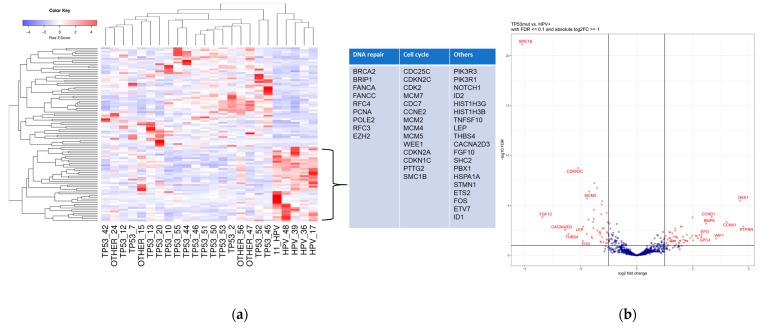
Depiction of the significantly differentially expressed genes. (**a**) Heatmap depicting the expression profiles of the genes that are significantly differentially expressed, |log2FC| ≥ 1.0 and FDR ≤ 0.1, between the two cohorts. The genes and samples were hierarchically clustered, according to the expression profiles, using complete linkage clustering. The corresponding dendrograms are shown on the left and at the top of the plot, respectively. The table includes all 42 genes that are significantly higher expressed in HPV+ vs. TP53mut VSCC. A complete list of all genes (upregulated, downregulated, and not differentially expressed) and the corresponding analysis results are provided in Table S2. (**b**) Volcano plot depicting the change of expression of genes with their significance for the comparison of TP53mut and HPV+ cohorts. Significantly differentially expressed genes are shown in red. The eight genes with the greatest positive and the eight genes with the greatest negative log2FC were labeled.

Among the 24 analyzed samples, four tumors were excluded from the TP53mut and HPV+ cohort and were termed “OTHER” (Table 1, for individual patient characteristics see Table S3). Two of them (24 and 47) were excluded due to TP53 wildtype (wt) status and the absence of HPV E7 integration. The two remaining samples, originally labelled as HPV+ tumors were reevaluated and subsequently excluded. In particular, sample 56 showed very few copies of E7 integration and displayed an oncogenic CCND1 mutation in WES, which is more likely to be the oncogenic driver in this tumor sample with HPV as a bystander infection. The other sample (15) was excluded, because the principal component analysis (PCA) revealed that its expression profile was more similar to that of the excluded samples 24 and 56 than to those of the other HPV positive samples. While the four aforementioned tumors (15, 24, 47, and 56) were excluded from the statistical analysis, we nonetheless included their corresponding gene expression profiles in Figure 1a.

### 3.2. Differential Expression in Subgroups

There were 98 genes significantly differentially expressed in primary TP53mut vs. HPV+ VSCC (Figure 1a,b). In the TP53mut cohort, 56 genes were upregulated and 42 were downregulated in comparison to HPV+ tumors (complete list of genes Table S2).

#### 3.2.1. TP53mut VSCC

In the TP53mut subgroup, Dickkopf-1 (DKK-1: log2 FC: 3.69) and cell cycle regulators CCND1 (log2FC: 2.68), CCND2 (log2FC: 1.82), CCNA1 (log2FC: 3.22), and CDK6 (log2FC: 2.25) were observed to be highly upregulated compared to HPV+ VSCC. Further significantly upregulated genes in the TP53mut subgroup with a log2FC greater than 1.25 and a normalized expression (average value of all samples) greater than 100 included LAMA1/3, WIF1, FZD3, MMP7, GRIA3, RAC2, THBS1, GPC4, IRS1, CSF3R, and CREB3L1, collagens as extracellular matrix components (COL1A1/COL1A2, COL3A1, COL5A, and COL5A2), PLCB4, FN1, INHBB, IL7R, BNIP3, and PDGFRA. See Figure 1b for the genes with the highest absolute FC, independent of their base mean.

**Figure 2:**
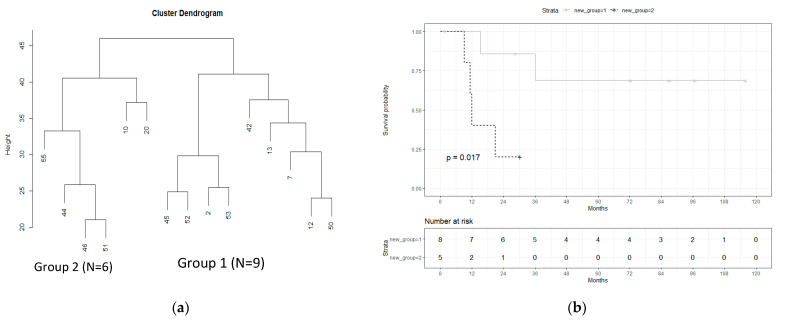
Subgroup analysis of the TP53mut group only. (**a**) Unsupervised hierarchical clustering of the TP53mut samples of which mRNA data was available (*n* = 15). (**b**) Kaplan−Meier curves and log rank test of the overall survival of the two groups identified by hierarchical clustering (*n* = 13, follow-up data of 2/15 cases were not available).

In the second step, the differential gene expression was evaluated within the TP53mut group. Using unsupervised hierarchical clustering, the TP53mut samples were subsequently separated into two groups, showing a significant differential expression of 20 genes (Figure 2a). Here, 10 genes were upregulated (TMPRSS2, IL20RA, RASAL1, MAPK8IP2, PLA2G3, PRMT8, HES5, SYK, IL1R2, and PLA2G4A) and 10 genes were downregulated (TSLP, PTPRR, IGFBP3, PRLR, ITGB3, FN1, VEGFC, DUSP10, GPC4, and RET) in group 2 in comparison to group 1 (Table 2). The highest log2FoldChange was shown in TMPRSS2, PLA2G3, IL1R2, PTPRR, TSLP, and GPC4, respectively.

The correlation with clinical parameters (pT, pN, tumor diameter, invasion depth, grading, recurrence, and survival) revealed a significant difference in overall survival between the two groups, showing a decreased overall survival (OS) in group 2 vs. group 1 (*p* = 0.017, Figure 2b). The median OS of group 2 was 12 months, whereas >50% of the patients of group 1 lived longer than the study’s follow-up. The correlation of all other clinical parameters (age, recurrence, pT/pN status, and tumor size) did not yield any significant results.

#### 3.2.2. HPV+ VSCC

In the HPV+ subgroup, the genes involved in cell cycle regulation were particularly often upregulated in comparison to the TP53mut tumors. In the heatmap where HPV+ VSCC cluster together (Figure 1a), the genes involved in G1/S transition, like CDKN2A, CCNE2, CDKN1C, and CDKN2C, as well as S/G2 and G2/M phase regulators CDK2, Wee1, CDC25C, and CDC7, and mini chromosome maintenance complex genes (MCM2/4/5/7) were particularly often upregulated. In addition, genes involved in DNA repair, like BRCA2, BRIP1, FANCA, FANCC, RFC4, PCNA, POLE2, RFC3, and EZH2, were upregulated compared to TP53mut tumors. Other significantly upregulated genes in the HPV+ cluster are shown in Figure 1a,b. None of the HPV+ tumors showed upregulation of cell cycle regulator cyclin D1, which were exclusively upregulated in the TP53mut tumors.

**Figure 3:**
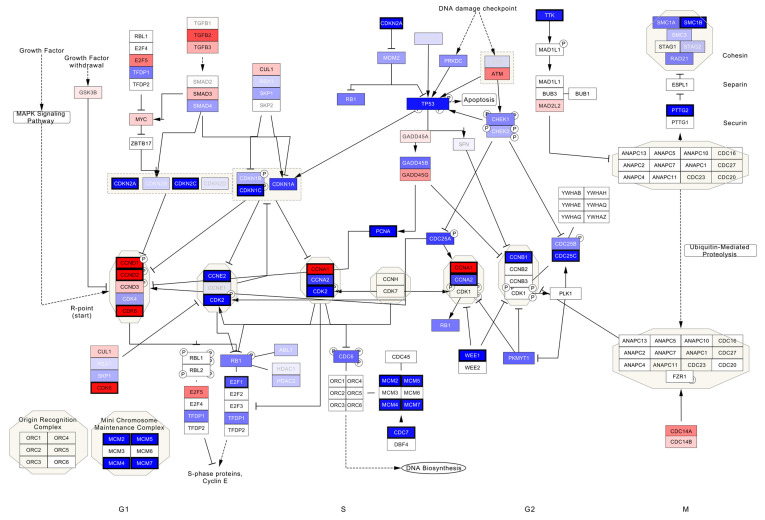
The human cell cycle pathway (WP179). The labels are colored according to the direction of the change in expression. Genes with a higher expression of TP53mut in comparison to HPV+ VSCCs are colored red. A thicker black border marks significantly (FDR ≤ 0.1) differentially expressed genes.

Pathway analysis confirmed the gene expression differences in different cell cycle control pathways (Figure 3; G1/S phase transition, G2/M checkpoints), as well as DNA repair.

No significant differences with recurrence, pT/pN status, tumor size, or survival were found between the HPV+ vs. TP53mut group.

#### 3.2.3. “OTHER”

**Figure 4:**
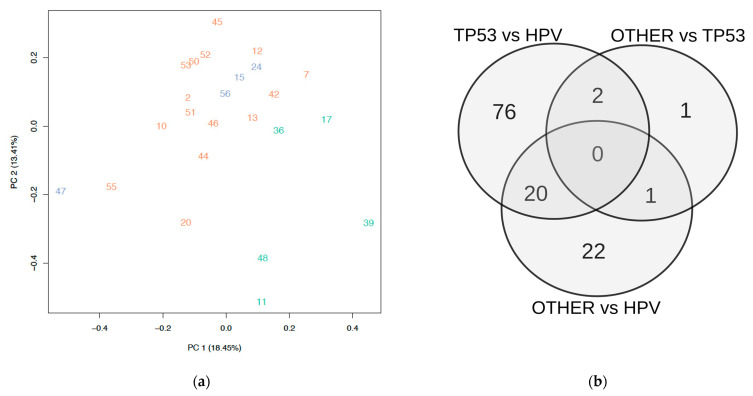
(**a**) Principal component analysis (PCA) of the three groups. Red: TP53mut, green: HPV+, blue: “OTHER”; (**b**) VENN diagram of the differentially expressed genes within the three subgroups; Detailed information on the underlying gene expression data and the intersections shown are provided in the supplements (Table S4).

Of the four samples that were grouped as “OTHER”, we observed that three (15, 24, and 56) clustered together in PCA (Figure 4a and Table S4). This small group of three samples showed more similarities in comparison to the TP53mut group than with HPV+ VSCC. Forty-three genes were differentially expressed in the comparison of this group and the HPV+ group, whereas four genes (CDC7, DNMT3A, ITGB8, and SMC1B) were differentially expressed between this and the TP53mut group. Only one gene (DNMT3A) was differentially expressed (downregulated) in comparison to both other subgroups (TP53mut and HPV+ VSCC).

## 4. Discussion

In the present analysis, the expression profiles of TP53mut VSCC were compared with those of HPV induced VSCC in order to characterize these two subtypes further. The HPV+ cohort showed significantly more differentially expressed genes in cell cycle regulation and DNA repair in the mitotic G1 phase, G1/S phase transition, and G2/M checkpoints. Besides *DKK-1*, which has been linked to a poor prognosis and to cancer progression, as well as displaying the highest log2FC in comparison to HPV+ VSCC, TP53mut tumors showed upregulation of some important key players of cell cycle regulation, e.g., *CCND1, CCND2, CCNA1,* and *CDK6*. However, no other commonly dysregulated pathways were identified in TP53mut VSCC. Within the *TP53* group, two subgroups with a significant differential expression of 20 genes were identified that correlated with OS.

As expected, a high expression of *CDKN2A* (p16) was observed as a surrogate for HPV induced transformation in the HPV+ cohort. Dysregulation of cell cycle and DNA repair mechanisms has been linked to an overexpression of the viral oncogenes E6/E7. They bind and thus inactivate tumor suppressors p53 and retinoblastom-protein (Rb), leading to uncontrolled proliferation. Whereas upregulation of cell cycle regulators *CDKN2A, CDKN1C, CDKN2C, CCNE2, CDK2, Wee1,* and *CDC7*, and mini chromosome maintenance complex genes *(MCM2/4/5/7)* was detected in the HPV+ cohort, upregulation of critical gatekeepers like *CCND1, CCNA1, CCND2,* and *CDK7* in cell cycle regulation was also observed in the TP53mut group. However, relevance for the tumor suppressor gene *CCNA1* has been reported in other HPV induced cancer, e.g., cervical and HNSCC. *CCNA1* promoter hypermethylation results in a decreased expression of cyclinA1 mRNA and thereby in a loss of function of the protein [31]. Evidence suggests that E7 of HPV is able to induce *CCNA1* promoter methylation by forming a complex with *Dnmt1* [32]. In a recently performed genome-wide methylation sequencing on a set of 18 VSCC and 6 normal vulvar tissue, 199 genes were found to be differentially methylated, without *CCNA1* being among them [19]. An explanation for this might be the small sample size of HPV induced cancers (*n* = 3) in their cohort.

*CCND1* is considered an oncogene and encodes for the cyclin D1 protein that regulates the transition through the restriction point in the G1 to S phase, thereby promoting proliferation and growth, as well as resistance to chemotherapy and radiotherapy, and a shorter OS [33,34]. Recently, several studies have shown that *CCND1* amplification is associated with a decreased response to immune checkpoint inhibitors (ICI) [35,36]. In a comprehensive analysis of three large databases (TCGA, Memorial Sloan Kettering Cancer Cancer (MSKCC) databases, and Geneplus cohort), it was revealed that *CCND1* amplifications in patients that receive ICIs is associated with decreased OS in different cancer entities. [37]. Furthermore, different cancer hallmarks were associated with *CCND1* amplification, including G2M checkpoints, p53 pathway, epithelial–mesenchymal transition, *(PI3K)/AKT*/mammalian target of rapamycin (mTOR) signaling, *KRAS* signaling, transforming growth factor (TGF)-b signaling, phosphoinositide 3-kinase, and hypoxia signaling in the pan cancer cohort of the TCGA. Interestingly, HNSCC that share distinct biological characteristics with vulvar cancer show a particularly high occurrence of *CCND1* amplifications (31%) in the TP53mut subgroup [20]. Other frequently amplified genes in the TCGA head and neck cohort, like *EGFR, FGFR1*, and *MYC,* were analyzed in our panel, but were not differentially regulated in TP53mut vs. HPV+ VSCC. The gene expression analysis from the *TCGA* database showed that *CCND1* amplification was significantly related to the upregulation of the mRNA expression of *CCND1* across the top nine cancer types. In our previous WES copy number analysis, significant differences were found in gain of chromosome 11, 67,000,001–71,000,001 bp between the TP53mut and HPV+ group [5]. This gain along chromosome 11 includes *CCND1,* and might be responsible for the gene’s overexpression. Cyclin D1 mutations are generally a rare event—only one somatic mutation was detected in our WES cohort. In a recently published targeted NGS analysis of 280 VSCC samples in 406 genes, Williams et al. demonstrated a high *CCND1* amplification rate in HPV− vs. HPV+ VSCC (22% vs. 2%; *p* < 0.001) [17]. In vulvar cancer, *CCND1* overexpression has been shown to be significantly related to the presence of regional lymph node metastases (*p* < 0.001) and HPV negativity (*p* < 0.001) [38].

In a recent RNA sequencing analysis published by Kolitz et al., VSCC were analyzed for differential expressed genes in HPV+ vs. HPV− tumors [23]. *E2F* was upregulated in HPV+ cases, following HPV E7 activation, and EYA2 was also upregulated, which has been shown to promote growth and migration in cervical cancer. In comparison to our own work, no overlapping expression patterns were detected. Differences in methodology, e.g., panel vs. whole transcriptome, subtype stratification (WES vs. polymerase chain reaction (PCR) and in situ hybridization (ISH)), and fresh frozen vs. paraffin embedded tissue preparation limits comparability.

Most of the differentially expressed genes reported by Kolitz et al. were not detected in our data and vice versa. However, STMN1 (Stathmin1) was also upregulated in HPV+ vs. TP53mut (log2FC: −1.04; base mean: 3254) VSCC in our analysis. Stathmin1 has previously been reported to be a highly sensitive and specific biomarker for the diagnosis of vulvar high-grade squamous epithelial lesion (HSIL) by Nooij et al. [39].

Activation of the *PIK3/Akt/mTOR* pathway by somatic mutation, *PIK3CA* copy number gains, or indirect regulatory effects such as the abrogation of cellular *mTOR* degradation via a loss of function of *FBXW7* has been reported across all NGS studies. It has predominantly been reported for HPV+ VSCC, but also in HPV- VSCC. Our expression data show a downregulation of *PIK3R1* and *PIK3R3* expression in TP53mut vs. HPV+ VSCC, encoding the regulatory isoforms p85α and p55γ of PI3K. Accumulating evidence also suggests that changes in p85α levels can modulate PI3K activation. It was reported that p85α depletion increases PI3K/AKT signaling and transformation in vitro, and accelerates tumor development [40]. A differential expression of FBXW7 was not observed in our analysis, however *NOTCH1*, one of its main targets, which it targets for degradation, was differentially expressed in our dataset. In 2017, a third HPV independent/*TP53wt* pathogenesis was first suggested by Nooij et al., later termed as “double negative” [41]. In a small cohort of 19 VSCC subjected to targeted NGS panel analysis, a high number of *NOTCH1* mutations were observed in HPV−/*TP53wt* subgroup (5/10). This subtype was not subject of our analysis, however, as we focused on differential expression between the so far clearly identified two prognostically relevant subtypes of VSCC. However, upregulation of *NOTCH1* was also observed in HPV+ vs. TP53mut VSCC in our cohort. *NOTCH1* is a transmembrane receptor involved in different cancer hallmarks like differentiation, proliferation, metastasis, apoptosis, and chemoresistance [42]. A variety of *NOTCH* inhibitors are being investigated in clinical trials, with a first phase III trial of inhibitor of the oral Nirogacesta, a γ-secretase inhibitor currently recruiting (NCT03785964). Our data add to the hypothesis of molecular heterogeneity among HPV independent VSCC. We observed that three of the four samples that were neither related to TP53mut nor HPV+ group show several similarities among them. In PCA, they are enclosed in a TP53mut subgroup and indeed show more similarities with TP53mut VSCC in terms of the gene expression pattern. Only *DNMT3A* was differentially expressed (downregulated) to TP53mut and HPV+ VSCC. *DNMT3A* belongs to a family of de novo DNA methyltransferases [43] and has traditionally been considered an oncogene, but can also act as a tumor suppressor under certain circumstances [44]. Just recently, hypermethylation of *DNMT3A* was reported in the above referenced genome-wide methylation sequencing in VSCC. Previously, an overexpression of *DNMT3A* was reported to be associated with increased risk of local recurrence in VSCC (HR = 4.5, *p* = 0.012), also in multivariate analysis after adjustment for disease stage (HR = 6.00, *p* = 0.003) and groin node metastasis (HR = 4.81, *p* = 0.008). Correlation with p16 staining revealed that the overexpression of *DNMT3A* was significantly correlated to p16 negativity. Whether there is clinical relevance in this *TP53wt*/HPV− vulvar cancer subgroup can only be speculated upon at this point and will need further validation.

In TP53mut VSCC, the highest expression of *DKK-1* was observed compared to HPV+ VSCC in our cohort. DKK-1 was originally described as a tumor suppressor by blocking the b-catenin dependent Wnt signaling pathway, which is frequently overactivated in cancer. More recently, elevated DKK-1 serum levels and protein expression were observed in a variety of tumor entities (e.g., gastric, breast, ovarian, and pancreatic cancer). Therefore, gene expression profiles were analyzed in the large TCGA cohort of 9677 tumor samples and 916 normal samples [45]. It was shown that DKK-1 mRNA was overexpressed in a wide range of tumors, e.g., head and neck, and pancreas, and was correlated with a shorter DFS. Early clinical study combinations of *antiDKK-1* Ab may improve the response to PD/PD-L1 therapy via the reduction of myeloid derived suppressor cells and upregulation in CD45+.

This analysis indicates that there might be evidence for a further subgroup within the TP53mut group that needs to be characterized in the future. Within the TP53mut group, two subgroups were identified with distinct mRNA expression patterns that correlated with survival. Nevertheless, the results should be interpreted with caution as the subgroups were very small in this cohort. The results should be confirmed in a larger independent cohort. The highest expression of *TMPRSS2, RASAL1, PLA2G3, HES5, IL1R2*, and *PLA2G4A* was shown in cluster 2 vs. cluster 1, and a lower expression was observed in *IGFBP3, FN1, VEGFC,* and *GPC4* in cluster 2 vs. cluster 1, respectively. To our knowledge, none of these genes have been linked to vulvar cancer pathogenesis so far. However, for some of the genes, clinical relevance has been shown in other tumor entities, e.g., in prostate cancer, an overexpression of *TMPRSS2* has been correlated to cancer cell invasion and metastasis [46], and a high *IL1R2* expression has been linked to a decreased overall survival and relapse-free survival in breast cancer patients [47]. *HES5* is a key regulator of *NOTCH* signaling and displays context-dependent oncogenic and tumor suppressive features in liver carcinogenesis [48].

Validation of these results in a larger second independent cohort including a subset of normal tissue will be the next step to confirm our results. As this analysis is focused on the differences between TP53mut vs. HPV+ VSCC, the results of the present expression analysis can only highlight the differentially expressed genes between the two subgroups. Expression changes that are relevant in both pathogenic pathways, like the *PIK3/Akt/mTOR* pathway, might therefore, not be identified.

## 5. Conclusions

RNA expression profiles show a distinct regulation of selective candidate genes with regard to the known VSCC subtypes and potentially enable further subclassification in the TP53mut group. For HPV+ VSCC, upregulation of the cell cycle regulators was predominantly seen. A small subset of three HPV−/*TP53wt* VSCC were classified as “other”, but were shown to share most RNA expression patterns with TP53mut rather than HPV+ VSCC.

## Figures and Tables

**Table 1:** Patient characteristics of HPV+ and TP53mut tumors. n.a., not applicable; FD, first diagnosis; yrs, years; SNL, Sentinel (individual patient characteristics are listed in Table S3).

Characteristics	HPV+ *N* = 5	HPV+ %	TP53mut *N* = 15	TP53mut %
Age at FD (yrs) median	63	n.a.	67	n.a.
(range)	(40–81)		(38–84)	
Tumor stage				
pT1b	1	20	3	20
pT2	4	80	8	53.3
pT3	0	0	1	6.7
pT4	0	0	1	6.7
unknown	0	0	2	13.3
Nodal status				
unilateral groin metastases	1	20	1	6.7
bilateral groin metastases	0	0	1	6.7
no groin metastases	4	80	10	66.7
unknown	0	0	3	20
Median tumor diameter				
mm	49.5	n.a.	50	n.a.
(range)	(45–50)		(15–150)	
Median depth of invasion		n.a.		n.a.
mm	10		7	
(range)	(8–10)		(5–28)	
Resection status of vulvar primary				
R0	3	60	11	73.3
R1	1	20	2	13.3
unknown	1	20	2	13.3
Grading				
G1	1	20	1	6.7
G2	2	40	5	33.3
G3	2	40	6	40
unknown	0	0	3	20
Vulvar surgery				
Vulvectomy	3	60	7	46.7
Radical local Excision	2	40	6	40
No local surgery	0	0	1	6.7
unknown	0	0	1	6.7
Groin Surgery				
Full Groin dissection (unilateral)	1	20	2	13.3
Full Groin dissection (bilateral)	4	80	5	33.3
SNL only (bilateral)	0	0	3	20
SLN^+^ followed by bilateral groin dissection	0	0	0	0
No LNE	0	0	4	26.7
Unknown	0	0	1	6.7
Pelvic node dissection	1	20	1	6.7
Adjuvant therapy				
Radiotherapy	2	40	3	20
Chemoradiation	1	20	0	0
Laser	0	0	0	0
no adjuvant therapy	2	40	11	73.3
unknown	0	0	1	6.7
Radiation field				
Vulva ±.groin ±.pelvis	3	60	2	13.3
Groin ±.pelvis only	0	0	1	6.7
unknown	0	0	0	0
Recurrence	1	20	3	20
Localization of recurrence (multiples possible)				
Vulva	1	20	3	20
Groins	1	20	2	13.3
Recurrence free	4	80	10	66.7
Distant metastasis in course of disease	1	20	2	13.3

**Table 2:** Significant differentially expressed genes between the two clusters within the TP53mut cohort, *p* < 0.001, +log2fold change higher gene expression in group 2 vs. group1. −log2fold change: lower gene expression in group 2 vs. group 1.

Gene	Log2FC	Base Mean	Gene Name
*TMPRSS2*	4.15	156.3	Transmembrane protease serine subtype 2
*PLA2G3*	2.66	136.03	Phospholipase A2 Group III
*IL20RA*	2.49	84.09	Interleukin 20 Receptor Subunit Alpha
*RASAL1*	2.45	156.73	RAS Protein Activator Like 1
*IL1R2*	2.18	526.73	Interleukin 1 Receptor Type 2
*PRMT8*	2.11	42.75	Protein Arginine Methyltransferase 8
*MAPK8IP2*	2.04	64.02	Mitogen-Activated Protein Kinase 8 Interacting Protein 2
*PLA2G4A*	1.95	127.75	Phospholipase A2 Group IVA)
*HES5*	1.58	125.22	Hes Family BHLH Transcription Factor 5
*SYK*	1.40	922.43	Spleen Associated Tyrosine Kinase
*PTPRR*	−3.05	55.63	Protein Tyrosine Phosphatase Receptor Type R
*TSLP*	−2.59	78.84	Thymic stromal lymphopoietin
*GPC4*	−2.25	269.24	Glypican 4
*IGFBP3*	−2.20	5798.71	Insulin-like growth factor-binding protein 3
*PRLR*	−2.08	67.63	Prolactin Receptor
*VEGFC*	−2.07	630.41	Vascular endothelial growth factor C
*FN1*	−1.9	6497.01	Fibronectin 1
*RET*	−1.67	75.33	Rezeptor-Tyrosinkinase
*DUSP10*	−1.25	543.2	Dual specificity protein phosphatase
*ITGB3*	−1.1	82.5	(Integrin Subunit Beta 3

## Data Availability

The data presented in this study are available in supplementary material.

## Supplementary Materials

The following are available online at https://www.mdpi.com/article/10.3390/cancers13246372/s1. Table S1 Somatic variants from whole exome sequencing analysis in vulvar cancer. Table S2: Results of the differential expression analysis (TP53mut vs. HPV+). Table S3: Individual patient characteristics of TP53mutated, HPV positive and “double” negative vulvar cancer. Table S4: Results of the differential expression analysis (“OTHER” vs. TP53mut and “OTHER” vs. HPV+) and intersections between all contrasts.
