# Supplementary material for: Transcriptome Analysis in Vulvar Squamous Cell Cancer
Source: Cancers (Basel). 2021 Dec 19;13(24):6372. doi: 10.3390/cancers13246372 (PMC8699756; doi:10.3390/cancers13246372)
Supplement: Supplementary file 1 [file cancers-13-06372-s001.zip › S3 invividual patient characteristics.pdf]

Supplementary Table S3. Individual patient characteristics of TP53mutated, HPV positive and 'double' negative vulvar cancer.

Tumor Samples	Clinical Parameters	*TP53* Mutation	HPV Status	Time to Recurrence (Months)	Time to Death (Months)
VSCC2	67yrs, vulvectomy, bilateral if-LNE, G1, pT2, pN0, G1, R0	TP53 missense variant (COSMIC)	negative	No recurrence/death after 116 months FU	
VSCC7	62yrs, vulvectomy, bilateral if-LNE, pT3, pN0, G2, R0, adj. radiation Vulva	TP53 missense variant (COSMIC)	negative	No recurrence/death after 97 months FU	
VSCC10	67yrs, vulvectomy, no if LNE, pelvic LNE, pT4, R1, cN0, adj. radiation to vulva and groins, G3, FIGO IVa	TP53 stop gained variant (COSMIC)	negative	Progressive disease	Death after 9 months
VSCC11	63yrs, radical local excision, unilateral if-LNE, pelvic LNE, pT2, pN0, G2, radiation vulva, R1	TP53wt	positive	n.a.	Death after 26 months
VSCC12	74yrs, radical local excision, no LNE, pT1b, pNx, R0, G2	TP53 missense variant (COSMIC)	negative	No recurrence/death after 0.5 months FU	
VSCC13	84yrs, radical local excision, no LNE, pT2, pNx, G3, R1, local recurrence, radiation	TP53 missense variant (COSMIC)	negative	Recurrence after 4 months	Death after 15 months
VSCC15	38yrs vulvectomy, bilateral if LNE, adj. radiation vulva, pT3, pN0, G3, R0	TP53wt	negative	No recurrence/death after 136 months FU	
VSCC17	81yrs, vulvectomy, bilateral if-LNE, radiation vulva, pT2, pN0, G3, R0	TP53wt	positive	n.a.	Death after 11 months
VSCC20	84yrs, vulvectomy, bilateral if-LNE, pT2, pN0, G3, R0	TP53 missense variant and stop gained variant (both COSMIC)	negative	n.a.	Death after 21 months
VSCC24	54 yrs, radical local excision, bilateral if-LNE, FIGO IVa, pT2, pN2, G2, R0,	TP53wt	negative	Progressive disease	Death after 15 months
VSCC36	49yrs, vulvectomy, bilateral if-LNE, pT2, pN0, G3, R0	TP53wt	positive	No recurrence/death after 54 months FU	

VSCC39	76yrs, vulvectomy, bilateral if-LNE, pT2, pN0, G2, R0,	TP53wt	positive	n.a.	Death after 18.5 months
VSCC42	42yrs, radical local excision, snl LNE, pT2, pN0, G2, R0,	TP53 splice donor variant (COSMIC)	negative	No recurrence/death after 87 months FU	
VSCC44	38yrs, radical local excision, unilateral if-LNE, pT2, pN0, G2, R0,	TP53 missense variant (COSMIC)	negative	No recurrence/death after 30 months FU	
VSCC45	46yrs, vulvectomy, pT2, cN0, G3, R0	TP53 stop gained variant (COSMIC)	negative	No recurrence/death after 2 months FU	
VSCC46	68yrs, vulvetomy, bilateral if-LNE, pT2, pN2, G2, R0, recurrence inguinal and vulva, radiation, distant metastasis in course of disease	TP53 missense variant (COSMIC)	negative	Recurrence after 5 months	Death after 11 months
VSCC47	94yrs, partial vulvectomy, pT2, pNx, G3, R0,	TP53wt	negative	No recurrence/death after 15 months FU	
VSCC48	40yrs, radical local excision, bilateral if-LNE, pT1b, pN1, G1, Rx, chemoradiation vulva and groins, recurrence vulva and groins, distant metastasis in course of disease	TP53wt	positive	Recurrence after 15 months	Death after 37 months
VSCC50	44yrs, radical local excision, snl only, pT1b, pN0, R0, G3	TP53 stop gained variant (COSMIC)	negative	No recurrence/death after 28.5 months FU	
VSCC51	68yrs, radical local excision, snl only, pT2, pN0, Gx, R0	TP53 missense variant (COSMIC)	negative		Death after 12 months
VSCC52	76yrs, vulvectomy, bilateral if-LNE, radiation groins and pelvis, pTx, pN1, G3, R0	TP53 missense variant (COSMIC)	negative	No recurrence/death after 72.3 months FU	
VSCC53	45yrs, no surgical primary therapy, primary radiation, unilateral if-LNE, cT1b, pN0, Gx, recurrence vulva and groins, distant metastasis in course of disease	TP53 missense variant and stop gained variant (both COSMIC)	negative	Recurrence after 11 months	Death after 36 months
VSCC55	No clinical information	TP53 missense variant (COSMIC)	negative	n.a.	n.a.
VSCC56	53yrs, vulvectomy, bilateral if-LNE, radiation to vulva, groins and pelvis, pT1b, pN0, G1, R0,	TP53wt	negative	No recurrence/death after 67.2 months FU	

Yrs: years; FU: follow-up, if-LNE: inguino-femoral Lymphadenectomy, snl: sentinel; wt: wildtype, FD: first diagnosis, n.a.: not applicable; TNM classification 6th edition was used [24, 25].
